# Limited Effectiveness of Psoralen- and Ultraviolet-Inactivated Vaccinia Virus on SHIV Infection

**DOI:** 10.3389/fimmu.2013.00327

**Published:** 2013-10-09

**Authors:** L. Lee Glenn

**Affiliations:** ^1^Institute for Quantitative Biology, East Tennessee State University, Johnson City, TN, USA

**Keywords:** HIV-1 vaccine, pathogenic SHIV, non-human primate, envelope cocktail, ultraviolet-inactivated vaccinia virus

The title and conclusions of the study recently published by Jones et al. (1) concluded that monkeys were protected from dying from a form of simian-human immunodeficiency virus (SHIV) by an psoralen- and ultraviolet-inactivated vaccinia virus in a multi-envelope DNA-VV-protein (DVP). However, the findings in the study are more equivocal than indicated by the title because the effectiveness of the modified vaccinia virus was not decisively demonstrated.

With regard to the effectiveness in reducing SHIV virus levels, the only significant effect was in reducing the *peak* viral loads, which was a fleeting condition around the second week after virus exposure. The more important virus levels are those over the long term because of the slow and progressive nature of SHIV. As the authors indicated, and which also is obvious from viewing the charts in Figure 4 from the above study, there was no significant action by DVP in the third through 17th week.

With regard to effectiveness of DVP in preventing reduction of T helper cells, the data in the study show that, with the exception of one case (monkey CA12), the envelope components alone (DNA and protein) appeared to offer protection that was comparable to the envelope components plus the psoralen- and ultraviolet-inactivated vaccinia virus (Figure 5). The idea that the envelope vehicle without the vaccinia virus might have been as effective as regular live vaccinia virus or DVP was not addressed.

With regard to preventing death, the findings do offer weak evidence for the notion that vaccinia virus delivered by any of the three methods (live, late, DVP, or possibly DNA/protein alone) have a protective effect because 66% of the unvaccinated cases died as compared to 6% of the vaccinated. The problem is that the statistical significance of the above difference in protective effect was based on Fisher’s Exact Test. If any cell has fewer than two or three cases, as established by Hanley and Lippman-Hand (2), there were zero cases for the cell for VV-protected cases that did not survive. A better statistical method for studying survival is Kaplan–Meier estimate which is designed for studying survival rates. When the data from the graphs in the figures of the above article were digitized and analyzed by the Kaplan–Meier method, the results are not statistically significant at *p* < 0.05 level. Thus, the conclusion that DVP had a protective effect against death is not supported at this time due to absence of statistical significance.

The study has much value because of the phenomenal amount of challenging biochemical and immunological work using a wide array of methods which each require expertise, the obvious passion for the development of a potent, readily manufactured vaccine against HIV, the new approach suggested of activating lymphocytes of many types, and other strengths. Nevertheless, due to over interpretation of the data, the main conclusion that DVP has a protective effect, and the implication in the title that the actions of DVP are different from the envelope or vaccinia virus by itself, are not warranted.
